# Neural Connectivity in Epilepsy as Measured by Granger Causality

**DOI:** 10.3389/fnhum.2015.00194

**Published:** 2015-07-14

**Authors:** Robert Coben, Iman Mohammad-Rezazadeh

**Affiliations:** ^1^NeuroRehabilitation & Neuropsychological Services, Massapequa Park, NY, USA; ^2^Integrated Neuroscience Services, Fayetteville, AR, USA; ^3^Semel Institute for Neuroscience and Human Behavior, David Geffen School of Medicine at University of California Los Angeles, Los Angeles, CA, USA

**Keywords:** epilepsy, connectivity, connectivity analysis, Granger causality, seizures

## Abstract

Epilepsy is a chronic neurological disorder characterized by repeated seizures or excessive electrical discharges in a group of brain cells. Prevalence rates include about 50 million people worldwide and 10% of all people have at least one seizure at one time in their lives. Connectivity models of epilepsy serve to provide a deeper understanding of the processes that control and regulate seizure activity. These models have received initial support and have included measures of EEG, MEG, and MRI connectivity. Preliminary findings have shown regions of increased connectivity in the immediate regions surrounding the seizure foci and associated low connectivity in nearby regions and pathways. There is also early evidence to suggest that these patterns change during ictal events and that these changes may even by related to the occurrence or triggering of seizure events. We present data showing how Granger causality can be used with EEG data to measure connectivity across brain regions involved in ictal events and their resolution. We have provided two case examples as a demonstration of how to obtain and interpret such data. EEG data of ictal events are processed, converted to independent components and their dipole localizations, and these are used to measure causality and connectivity between these locations. Both examples have shown hypercoupling near the seizure foci and low causality across nearby and associated neuronal pathways. This technique also allows us to track how these measures change over time and during the ictal and post-ictal periods. Areas for further research into this technique, its application to epilepsy, and the formation of more effective therapeutic interventions are recommended.

## Introduction

Epilepsy is a chronic neurological disorder characterized by repeated seizures or excessive electrical discharges in a group of brain cells. Prevalence rates include about 50 million people worldwide and 10% of all people have at least one seizure at one time in their lives (World Health Organization (WHO), 2012). The reported median incidence of epilepsy was 50.4/100,000 persons/year (Ngugi et al., 2011). Unfortunately, there is still a gap in our understanding of the neurophysiological mechanisms that lead to seizures and at least 30% of the population does not respond to the mainline treatment, medication. At a basic neurochemical level, seizures are thought to arise when the balance between excitation and inhibition is disrupted (Engel et al., 2013). At the heart of this is the notion that there is a disruption in neuronal communication or connectivity that then leads to excessive electrical discharges or a seizure. Studying the electroencephalogram is the most common method for seizure detection with varying accuracy rates across studies. In a recent analysis, seizure detection was at 84% with enhancements of accuracy rates with more advanced computer analyses of latency and wavelets (Ahammad et al., 2014).

## Connectivity Theory of Epilepsy

The earliest work in EEG described neurons as the units that transmit signals through synaptic connections and form the basis for neuronal networks (Ramón y Cajal, 1894). Jump ahead to more modern times and we have those that have proposed cortical and functional networks to explain the integration and outcome of these cellular paths (Mesulam, 1990; Abeles, 1991). Within this, there have been descriptions of “local networks” and more “large-scale networks” representing the differences in distance across these systems. Nodes that connect local networks and hubs that dominate the majority of pathways within larger networks have been described. It has been observed that local networks have hubs composed of neurons and glia that become increasingly coupled at seizure onset and thus incorporating more distant networks during this process (Zhang et al., 2011). In their important paper, Stefan and Lopes da Silva (2013) have described how epilepsies may be understood from a neuronal network perspective. They also describe how all epilepsies may be understood from a local or “focal” perspective and that more generalized seizures represent faster propagation pathways. It is what happens within the local network and surrounding regions that is critical to the formation of these events.

In cases of temporal lobe epilepsy, abnormalities of gray, white matter, and inter-regional fiber diffusivity have been found within and beyond mesiotemporal and temporo-limbic networks (Ahmadi et al., 2009). In addition, recent findings using graph theoretical approaches have shown disruptions of inter-regional and functional connections in such patients (Bonilha et al., 2012). In focal epilepsy, these networks are characterized by increased clustering and path length (Bernhardt et al., 2011). Interestingly, these network anomalies intensify over time and are more pronounced in surgical patients that continue to have seizures. When measuring resting state functional networks in such patients, there is evidence of both increased temporal and decreased frontal–parietal connectivity (Liao et al., 2010). Similarly, Maccotta et al. (2013) studied fMRI connectivity in a series of 32 patients with temporal lobe epilepsy. They showed decreased local coupling including regions of the mesial temporal lobe, parahippocampus, and hippocampus. In addition, there was also evidence of interhemispheric decoupling. Interestingly, there were also patterns of increased coupling within the ipsilateral insula and neighboring subcortical regions. Clemens et al. (2013) have also shown a combination of hypocoupled and hypercoupled connectivity in juvenile myoclonic epilepsy with greater alterations in connectivity occurring during the ictal state. Using a novel approach of voxel-based fMRI connectivity, Constable et al. (2013) have shown the presence of focal high connectivity, often involving the seizure foci, with associated low connectivity in nearby regions and pathways.

These preliminary findings are intriguing and must clearly be replicated over time and in multiple patient groups and for different forms of epilepsy. However, they do lead us to a theory of neural connectivity underlying the formation of seizures. Within this theory, we postulate regions of hyper- and hypo-connectivity impacting seizure propagation and the likelihood of such events. Hypercoupling seems to be present surrounding the seizure foci setting a tipping point for coupled and hypersynchronized discharges as are seen in seizure events. Surrounding this are regions of hypocoupling that may help to regulate the likelihood of an event occurring. We have recently shown that coherence across these regions undergoes a significant hypocoupling preceding and at the initiation of spike and seizure occurrence. Tools that measure these changes over time are critical and may facilitate understanding of these processes.

Granger causality (GC) estimates of connectivity in epilepsy have been shown to possess some validity. It has shown similar results to dynamic causal modeling (David et al., 2008), has plausible estimates of human seizure propagation pathways (Murta et al., 2012) and has been in line with pathways demonstrated with DTI as well (Bhardwaj et al., 2010). This suggests that there are initial findings indicating that GC methods of connectivity may validly depict pathways important for seizure propagation.

## Effective Connectivity as Measured by Granger Causality

An advanced statistical technique for investigated directed causation that uses multiple autoregressive (AR) analyses is GC and its related concepts of partial directed coherences (PDCs) (Seth, 2010). Granger causality analysis (GCA) is a method for investigating whether one time series can correctly forecast another (Bressler and Seth, 2010). GC is a data-driven approach based on linear regressive models and requires only a few basic assumptions about the original data statistics. Recently, in neuroscience applications, GC has been used to explore causal dependencies between brain regions by investigating directed information flow or causality in the brain. It uses the error prediction of AR or multi-variant autoregressive (MAR) models to estimate if a brain process is a Granger-cause of another brain process.

## Connectivity Analyses

The goal of any connectivity analysis in the brain is to understand how the brain’s circuits are structured and how they change during time (Kispersky et al., 2011). Three types of brain connectivity can be defined to understand the brain’s circuits and interactions between different parts of the brain: anatomical, functional, and effective connectivity (Liu and Aviyente, 2012). Anatomical connectivity techniques rely on physical and structural connections of neuronal units. However, and especially in non-adult populations, the number of well-established brain circuits is low and on the other hand the connectivity diagram provide static description of brain connectivity. The main aim of functional connectivity analyses is to find temporal or spatial statistically co-variation of neuronal units’ activities, which typically reveals cross-correlograms or coherency measures; however, in these kinds of analyses the magnitude and direction of information flow and interactions of different neuronal units are not considered. Effective connectivity analyzes the direction, interaction, and effect of a neuronal unit activity on other units in both time and frequency. Effective connectivity can be used to understand causal relationships between entities of the brain network and prediction of the activity of one brain structure by the incorporation of information from the past activities of other brain structures. As described in Vicente et al. (2011), the measures of casual relationships between network entities can be categorized into two large classes of methods: one class of methods measures the effective connectivity between entities of a network based on the amount of information that exists in random variables associated with network entities. The other class of methods basically try to model the whole process that generates the data for the network entities.

Because of the importance of effective connectivity and information flow analyses in the brain, in the rest of this section we will discuss the qualitative and quantitative definition of its popular definition, Granger casualty, and its derivatives and applications in EEG analyses.

## Granger Causality

By reviewing related literatures, it can be found that one of the most popular definitions for causality, which falls within modeling class of effective connectivity is GC. It was first introduced by Weiner in 1956 and later formalized by Granger in form of linear regression method in 1969 (Hu et al., 2012). GC can be simply defined as follows:

### Definition

Suppose two stochastic processes *X*_1_(*t*) and *X*_2_(*t*) and future values of *X*_1_(*t*) is going to be predicted by using two different data sets: using only the past values of *X*_1_(*t*) and by incorporation of past values of *X*_1_(*t*) and *X*_2_(*t*). If incorporating the past knowledge of *X*_2_(*t*) permits more accurate prediction of *X*_1_ then *X*_2_ could be called a casual to *X*_1_ (Cadotte et al., 2008).

#### Mathematical form of GC

Suppose *X*_1_ and *X*_2_ can be represented by single variable AR models:

$$\begin{array}{lll} X_{1}\left(t\right)=\sum_{\text{j}=1}^{\text{m}}a_{j}X_{1}\left(t-j\right)+\varepsilon_{\mathrm{11}}\left(t\right) & \; & \\ X_{2}\left(t\right)=\sum_{\text{j}=1}^{\text{m}}b_{j}X_{2}\left(t-j\right)+\varepsilon_{\mathrm{22}}\left(t\right) & \; & \end{array}$$

A joint predictor of *X*_1_(*t*) can be defined as:

$$X_{1}^{*}\left(t\right)=\sum_{\text{j}=1}^{\text{m}}a_{j}^{*}X_{1}\left(t-j\right)+\sum_{\text{j}=1}^{\text{m}}b_{j}^{*}X_{2}\left(t-j\right)+\varepsilon_{\mathrm{12}}\left(t\right)$$

Which is a part of the multivariate model of the “process” that generates *X*_1_ and *X*_2_. Here, if the variance of prediction error $\delta_{12}^{2}\left(\varepsilon_{\mathrm{12}}\right)$ is less than the variance of $\delta_{12}^{2}\left(\varepsilon_{\mathrm{11}}\right)$ then it is an indication of a causal interaction from *X*_2_(*t*) to *X*_1_(*t*). The magnitude of causality from *X*_2_ to *X*_1_ is defined as $F_{\mathrm{x2}\to \mathrm{x1}}\;=\;\ln\left(\frac{\delta_{12}^{2}}{\delta_{1}^{2}}\right)$; thus if $\delta_{1}^{2}\;=\;\delta_{12}^{2}$ then the magnitude of causality from *X*_2_ to *X*_1_ is 0. In a similar way, *F*_x1→x2_ the causality from *X*_1_ to *X*_2_ also can be defined as well. The asymmetry in *F*_x1→x2_ and *F*_x2→x1_ indicates the directionality of causality between *X*_1_ and *X*_3_ (Vakorin et al., 2013): Δ*F* = *F*_x2→x1_ – *F*_x1→x1_. If Δ*F* is positive then the net direction of causality (coupling) is from *X*_2_ to *X*_1_, and vice versa.

#### Conditional Granger causality

Now suppose a system with more than two variable time series. One question is whether in this system a causal influence between any pair of time series is directed or mediated by others (Dhamala et al., 2008). For example, if *X*_1_ has causal influence on *X*_2_ and *X*_2_ has causal influence on *X*_3_ then *X*_1_ has *indirect* causal influence on *X*_3_. Direct and indirect causation between two time series or in general two variables of a system can be defined by conditional GC $F_{\mathrm{x1}\to \text{x3}|\mathrm{x2}}=\ln\left(\frac{\delta_{1,3}^{2}}{\delta_{1,2,3}^{2}}\right)$ where $\delta_{1,2,3}^{2}$ is the variance of the error of predicting *X*_1_ using the past values of *X*_1_, *X*_2_, and *X*_3_.

#### Granger causality limitations

Granger causality needs three main prerequisites to be applied on brain data: (1) linear interaction model between network entities. Thus, GC has been mostly applied using a multivariate autoregressive (MVAR) model by incorporating methods such as direct transfer function (DTF), PDC, and directed partial coherence (DPC). These approaches may cause misleading results when applied in signals that have non-linear dependencies such as EEG; thus applying blind source separation (BSS) methods like independent component analysis (ICA) can be helpful to have maximally independent data sources before applying GC method on the data (Liu and Aviyente, 2012); (2) relatively low noise level in data. Again, data cleaning data from bad components using methods such as ICA can be beneficial to obtain cleaner data; and (3) low cross-talk between the measurements of network entities. To tackle this issue, which arises mostly because of the scalp volume conductance, GC analyses can be performed by applying GC on ICA brain sources or current source density (also known as Spatial Laplacians) signals instead of original brain voltages from scalp channels (Vicente et al., 2011; Coben et al., 2014).

### Practical notes

#### Sampling frequency

It should be noted that the accuracy of GC model prediction may increase when more input data is provided (Kayser et al., 2009) to it and thus having sufficiently high sampling frequency (more than 256 Hz) can be beneficial.

#### Re-referencing

As mentioned above, applying connectivity analyses to channel level data generally lead to some false positives coupling between the electrodes due to the volume conductance problem on the scalp. In general, an average reference or Laplacian reference are better choices than other referencing methods such as linked mastoid reference (Nunez et al., 1997).

#### Filtering data

Using causal filter may adversely effect the direction of information flow in the GC analysis. It is recommended that one use a non-causal filter (for example, finite impulse response filters) with zero phase lag (Mullen et al., 2012).

#### Model order selection

In our previous paper, it is mentioned how to select and validate a model order. Model order can be determined based on akaike information criterion (AIC) and Bayesian information criterion (BIC) criteria to maximize the model effects. In addition, the critical issue for GC is the ratio between the number of independent observations (i.e., samples) and the model complexity (i.e., model order or number of parameters). If the number of observations is large relative to the number of parameters then model order selection criteria are most likely valid. If the number of observations is small, then other criteria such as corrected AIC should be used for model order selection. Increasing the number of sources to analyze in the same time in a GC analysis may lead to more computational cost, higher model order and, as a result, a reduction of the model accuracy if the number of samples are fixed.

#### Model stationarity and stability

A process or model is strongly stationary if its probability distribution is time invariant. If only its first and second moments are time invariant then the process is called weakly stationary. In mathematical terms, if all the eigenvalues of the module of roots of poles in a MVAR model are less than one then the model is stationary. If drift is present on data it is recommended to detrend data before fitting a MVAR model on it because trend brings non-stationarity to the MVAR model. A MVAR model is stable if its reverse model has no roots in or on the complex unit circle; in other words its response is bounded for bounded input, which means it has finite impulse response. In general, a stable MVAR model is always stationary and thus if the model passes stability and whiteness tests (e.g., the data can be appropriately modeled as a stable VAR process), stationarity of the data is inherently implied. However, in cases where the model residuals are not white or the model is not stable, it can be useful to run a multivariate stationarity test on the data to determine if this is the problem.

#### Causality between independent sources from ICA – a contradiction!

It could be a common question to ask when using ICA it is assumed that there is mutual independence between underlying sources, however, when the connectivity between EEG sources is estimated, it is implicitly assumed that the sources may be influenced by each other. This contradicts the fundamental assumption of mutual independence between sources in ICA. However, causality (Granger or any other causality algorithm for that matter) states that there is causality between past information of the source of influence and the current information of the recipient of influence. It should be noted ICA essentially tries to eliminate instantaneous dependence between signals at each time point. Therefore, causality and ICA do not contradict (at least, conceptually). In general, any BSS algorithm is also conceptually similar to ICA since it minimizes the instantaneous dependence between signals. In other words, IC sources are “independent” at time point *t* but ICA does not guarantee to remove any dependency between two different processes or signals at different time points (*t* and *t* + *m*, for example).

Here, we would want to elaborate more about the above question using a traditional example: *Cocktail party*. Suppose that John and Mary and other people are in a party and John asked Mary “How are you?” and Mary replied “I am fine, yourself?” A listener from a distance could hardly hear the communication because of a lot of noise from people in the environment. If ICA is applied to the sounds properly then it is possible to *separate* John’s, Mary’s, and other people’s voices. Now, there are three independent (separate) sources but Mary’s dialog is correlated with John’s dialog or more precisely John’s dialog is a causal factor of Mary’s dialog. Thus, being independent does not imply being uncorrelated or non-causal.

## Granger Causality of EEG Seizure Data: Case Studies

**Figure 1:**
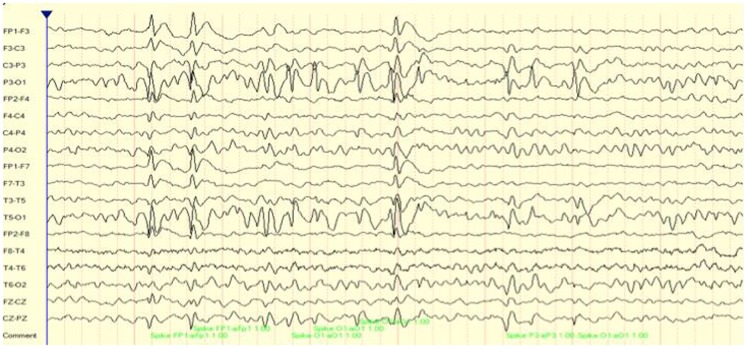
**EEG (longitudinal/sequential montage) sample showing focal (T5, P3) spike and wave discharges for case 1**.

For demonstration purposes, we present two cases of focal seizure disorders. Case 1 is an 8-year-old boy who presented with limited communication skills, poor auditory comprehension, and an inability to read or identify letters. As a result, his academic and social skills were impacted tremendously. As may be seen in Figure 1 his EEG included multiple focal spike and wave discharges that predominated over the surface between T5 and P3 (left temporal–parietal regions). Over a 16-min recording time there were 225 of these events, one every 4 s. This is interesting clinically as this region is critical for language comprehension and reading of words. For subsequent analysis, an EEG record was created composed of these multiple events only. In essence, we are looking at connectivity patterns during and surrounding the ictal events.

**Figure 2:**
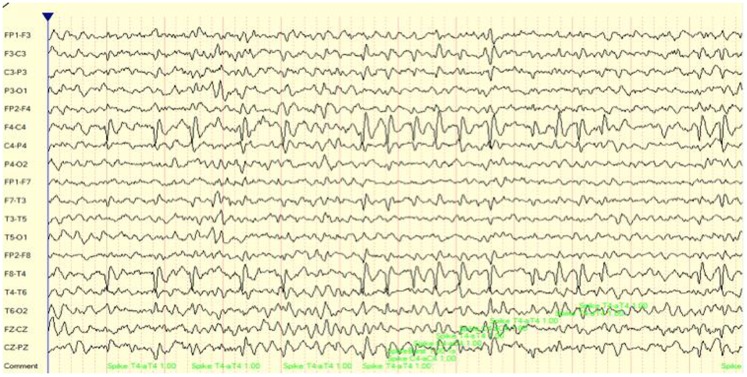
**EEG (longitudinal/sequential montage) sample showing focal (F4, T4) spike and wave discharges for case 2**.

Case number 2 is of a 9-year-old girl on the autistic spectrum who presented with a partial seizure disorder over the right hemisphere. This has been associated with cognitive and attention deficits as well as significant social skill challenges. As may be seen in Figure 2, there were multiple, repeated focal spike and wave complexes over right frontal and temporal regions. In fact, over a 15-min recording time there were 295 such events detected by the Persyst 12 seizure/spike detection algorithm. This equates to a spike complex occurring every 3 s.

### Methods

EEG data were collected under two conditions, eyes closed and eyes open, with the eyes open forming the basis for these analyses. A stretchable electrode cap embedded with 19 sensors attached to the scalp was used to collect data, with frontal reference, prefrontal ground, and linked ears. Each recording lasted 20 min, where 10 min were spent in both conditions. This included recording and digitizing EEG readings based on the International 10/20 System of electrode placement utilizing the Deymed Diagnostic (2004) TruScan 32 Acquisition EEG System. This system included 32 channels with sampling at 128 cycles per second and filtering between 0.1 and 40 Hz. All recordings were done with impedance <5 KΩ. The common mode rejection ratio for this system is 102 dB and the isolation mode rejection ratio is 140 dB.

To perform such an analysis, we utilized the source information flow toolbox (SIFT) toolbox from EEGLAB v.12 (Delorme et al., 2011) on EEG data from two patients manifesting seizure activity. A key aspect of SIFT is that it focuses on estimating and visualizing multivariate effective connectivity in the source domain rather than between scalp electrode signals. This should allow us to achieve finer spatial localization of the network components while minimizing the challenging signal processing confounds produced by broad volume conduction from “neural” sources to the scalp electrodes. From EEG data we have virtually epoched this stream into 1-s segments. ICA was then used to extract unique, independent components from the data. To fit multiple component dipoles and determine their locations DIPFIT toolbox was then applied. Then by investigating the dipole locations and the components topographical maps, only good “neural” components that are related to neural process in the brain have been included for further processing. These data were then fit into a MAR model using Vieira–Morf algorithm. For our data, the model and after some trials and errors and model validation process, the MAR model order has been set to 5. In addition, the frequency band of interest has been selected from 1 to 30 Hz and the most obvious connectivity measure was Grager–Geweke causality (GGC).

To quantify the results of GC analyses the magnitude of the average value for all GC values across the frequency range of interest (1–30 Hz) was calculated and defined as average GC. The value of *f*_1_ and *f*_2_ depend on the required frequency resolution. In our study, hyper- and hypo-connectivity is defined by the magnitude of GC. In future studies and especially to investigate (de)synchronization in the brain, the phase of GC values will be also considered. Choosing too wide a frequency window to average may eliminate some effective connections and reduce the accuracy of the measurement.

$$\mathrm{Average}\;\mathrm{Causality}\;\left(t\right)=\frac{\sum_{\mathrm{freq=f1}}^{\mathrm{freq=f2}}F_{\text{x}2\to \text{x}1}\left(\mathrm{time,freq}\right)}{f2-f1}$$

**Figure 3:**
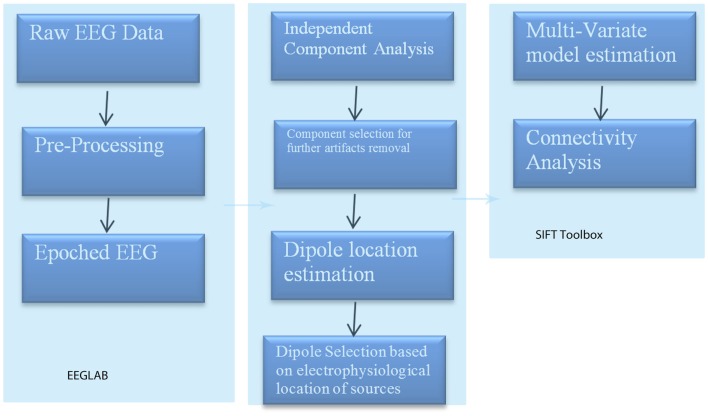
**SIFT/Granger (GGC) causality sequence of processing**.

These methods of operation are summarized in Figure 3. This takes the EEG data from sensory to source space via ICA and dipole localization. This diminishes the issue of volume conduction [see Astolfi et al. (2007) and Akalin Acar and Makeig (2013)]. Once dipole localization has been performed, these data are subjected to MVAR and GC analysis as presented above. Within a reasonable range of values, changes in model order may show little effect on the spectral density (and by extension coherence) [e.g., see Florian and Pfurtscheller (1995)]. Our model order has been based on AIC and BIC criteria to maximize model effects. Statistically, the critical issue for GC is the ratio between the number of independent observations (i.e., samples) and the model complexity (i.e., number of parameters). If the number of observations is large relative to the number of parameters then the model order selection criteria are still valid. If the number of observations is small, then we might run into problems with AIC and other asymptotic estimators, but there are corrections for that (corrected AIC). In our data set (case epoching), we have plenty of data available and the ratio of observations [total data samples within a time window (× trials)] to parameters is >40 suggesting that we have a valid model using AIC (Burnham, 2004).

## Case Examples: Results and Analyses

Following the methodology reviewed above and shown in Figure 3, eight components were selected with their dipoles being localized to the following regions: right superior frontal gyrus (Broadman 6), left superior occipital gyrus, right medial frontal gyrus white matter, left thalamus, left middle frontal gyrus white matter, left precentral gyrus, right superior frontal gyrus (Broadman 9), and the left middle temporal gyrus. SIFT/GCA was then performed with and between these source localized regions. As may be seen in Figure 3 greater coupling is revealed over the left superior occipital gyrus and its influence on the left middle temporal gyrus and additionally over the right superior frontal gyrus. Maximal regions of lower connectivity are observed between the left middle temporal gyrus, left precentral and middle frontal white matter, and the left thalamus.

Previous studies have focused on various cortical areas and thalamic nuclei in the generation of absence seizures. Tenneya et al. (2013) used beamformer analysis using synthetic aperture magnetometry (SAM) to confirm the presence of independent thalamic activity. They found that sources detected in the 50 ms prior to the start of the seizure were more likely to be localized to the frontal cortex or thalamus. However, the ability to detect independent signals within the cortex and thalamus is an important factor to assign relative contributions of each source to seizure activity (Gupta et al., 2011). ICA can be a promising algorithm to separate individual statistically independent sources. One limitation could be the spatial resolution of electrodes on the scalp, which plays a key role on the accuracy of source locations. The comparison about the performance of available source localization methods is beyond the scope of this study. However, the present study is one of the few studies that investigate the causality and information flow between different possible sources of seizure using EEG data in a clinical population. Future studies will be conducted on more subjects and group level analyses will be also performed to find out whether there is any common pattern of activities and loci among possible sources before or during seizure activity.

**Figure 4:**
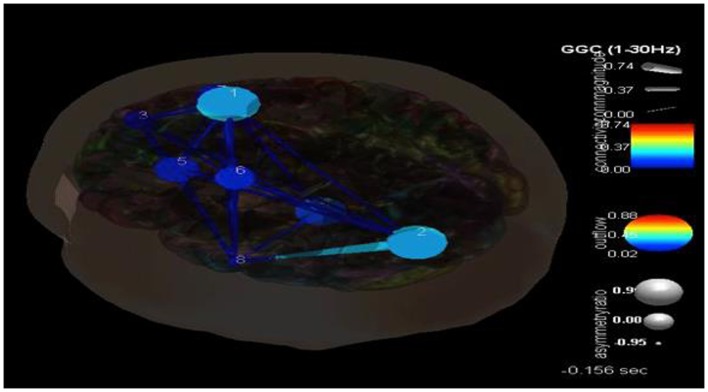
**SIFT/Granger causality baseline image (case 1) for eight component dipoles localized to the right superior frontal gyrus (Broadman 6), left superior occipital gyrus, right medial frontal gyrus white matter, left thalamus, left middle frontal gyrus white matter, left precentral gyrus, right superior frontal gyrus (Broadman 9), and the left middle temporal gyrus**. Smaller, dark blue circles and dark blue, thin lines indicate lower causality. This causality image at baseline (pre-ictal) occurs at −0.156 s.

**Figure 5:**
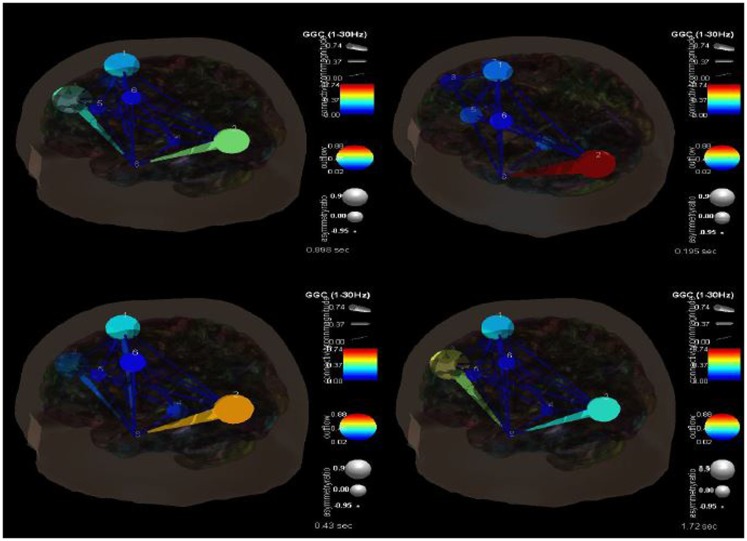
**SIFT/Granger causality baseline image (case 1) for 8 component dipoles and how they change over time**. The sequence goes from 0.089 s (top left) to 0.195 s (top right) to 0.42 s (bottom left) to 1.72 s (bottom right).

One of the advantages of this type of analysis is that it can show causality and how it changes over time. Figure 4 shows the transition in causality as these focal spike and wave discharges evolve over time from the ictal through the post-ictal phase. It becomes even more clear that there is a hypercoupling involving the left superior occipital gyrus and its influence over the left middle temporal gyrus. This is the same region over which these events appear at the surface and may be considered the foci or focal region(s). As these events progress (Figure 5), these regions’ hypercoupling lessens but remains to a greater degree than the other pathways. The hypoconnected regions involving the pathways between the left temporal gyrus, left frontal white matter, left precental gyrus, and left thalamus have the lowest causality during the ictal peak and then “recover” somewhat into the post-ictal period. Interestingly, it also seems that the superior frontal gyrus exerts some causality over the left middle temporal gyrus during the recovery period. This may stabilize the event and prevent propagation further. Clearly, these are hypothesizes that would need to tested further and seen in repeated cases before that could be accepted as factual.

**Figure 6:**
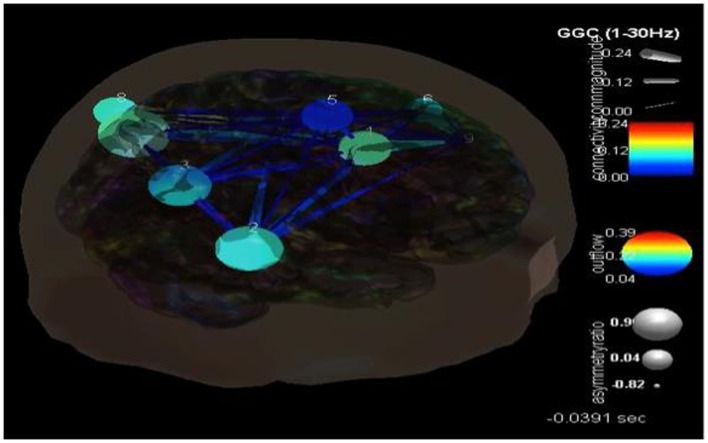
**SIFT/Granger causality baseline image (case 2) for nine component dipoles localized to the right anterior cingulate gyrus (Broadman 32), right superior temporal gyrus (Broadman 22), right precuneus white matter, left postcentral gyrus (Broadman 43), right medial frontal gyrus white matter, left middle frontal gyrus (Broadman 46), left precuneus (Broadman 39), left supramarginal gyrus (Broadman 39) and the left middle frontal gyrus white matter**. Smaller, dark blue circles and dark blue, thin lines indicate lower causality. This causality image at baseline (pre-ictal) occurs at −0.0391 s.

For case 2, ICA component analysis of this seizure data revealed 9 independent components. Their dipoles were source localized to the following regions: the right anterior cingulate gyrus (Broadman 32), right superior temporal gyrus (Broadman 22), right precuneus white matter, left postcentral gyrus (Broadman 43), right medial frontal gyrus white matter, left middle frontal gyrus (Broadman 46), left precuneus (Broadman 39), left supramarginal gyrus (Broadman 39), and the left middle frontal gyrus white matter. SIFT/GCA was then performed with and between these source localized regions. As may be seen in Figure 6 there appears to be enhanced coupling involving the precuneus and supramarginal gyrus and additionally over the right anterior cingulate gyrus. Low causality is seen in the right medial frontal gyrus white matter and in the pathways/connections between this, the anterior cingulate, right superior temporal gyrus, and right precuneus white matter.

**Figure 7:**
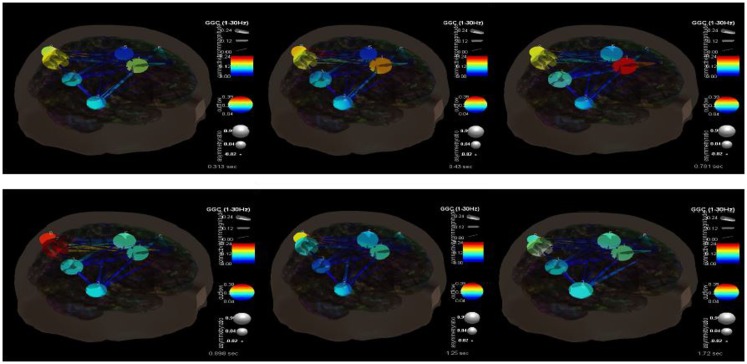
**SIFT/Granger causality baseline image (case 2) for nine component dipoles and how they change over time**. The sequence goes from 0.313 s (top left) to 0.43 s (top middle) to 0.781 s (top right) to 0.898 s (bottom left) to 1.25 s (bottom middle) to 1.72 s (bottom right).

Figure 7 shows the changes in causality over the time course of these ictal events. It is clear that during the beginning ictal phase that the right anterior cingulate becomes more hypercoupled with the medial frontal gyrus and its white matter connections. We would theorize that this is the seizure foci that becomes hypersynchronized during these events. As the ictal period ends and moves into a post-ictal phase this hypercoupling relaxes and returns to baseline. During this transition there also appears to be an interesting hypercoupling of the posterior precuneus, supramarginal gyrus region, and its white matter pathways. This is also associated with an increase in causality from posterior to anterior regions. There is also a pervasive pattern of low connectivity at and between regions including the right superior temporal gyrus, precunues white matter, frontal medial gyrus white matter, postcentral gyrus, and the anterior cingulate. The causality across these regions appears to decrease at seizure initiation and only increases once again near its completion. It is clear that there are multiple, very complex connectivity relationships involved in these processes that need to be understood better.

## Discussion

Connectivity models of epilepsy serve to provide a deeper understanding of the processes that control and regulate seizure activity. These models have received initial support and have included measures of EEG, MEG, and MRI connectivity (Ahmadi et al., 2009; Bonilha et al., 2012; Stefan and Lopes da Silva, 2013). Preliminary findings have shown regions of increased connectivity in the immediate regions surrounding the seizure foci (Liao et al., 2010; Maccotta et al., 2013) and associated low connectivity in nearby regions and pathways (Constable et al., 2013). There is also early evidence to suggest that these patterns change during ictal events and that these changes may even be related to the occurrence or triggering of seizure events (Bernhardt et al., 2011). As a means to measure these processes GC estimates of connectivity in epilepsy have been shown have some validity. It has shown similar results to Dynamic Causal Modeling (David et al., 2008), has plausible estimates of human seizure propagation pathways (Murta et al., 2012), and has been in line with pathways demonstrated with DTI as well (Bhardwaj et al., 2010).

Mathematically, ICA does not have any problem to extract different independent sources even if they are all synchronized. Thinking about a performance in a concert and instruments, which are played by different musicians, ICA can separate out each sound from a specific instrument even if all instruments are played in harmony and synchronized. However, in our future approach, the functional connectivity between affected regions will be investigated using features from graph theory such as assortativity, clustering coefficient, local, and global efficiency. This new approach will enable us to investigate and quantify the severity of the multi-focal activities, the connectivity pattern between different regions, and the effect of taking medications and therapy on the connectivity pattern (Mégevand and Vulliémoz, 2013).

We have provided two case examples as a demonstration of how to obtain and interpret such data. We have shown the results of a pilot study. The feasibility and limitations of the proposed methods have been investigated. For future studies, we recommend more subjects, in-depth statistical analyses and group-based comparisons. EEG data of ictal events are processed, converted to independent components and their dipole localizations, and these are used to measure causality and connectivity between these locations. Both examples have shown hypercoupling near the seizure foci and low causality across nearby and associated neuronal pathways. This technique also allows us to track how these measures change over time and during the ictal and post-ictal periods. Interestingly, in both examples, there is some increased frontal connectivity as the recovery period ensues. One might theorize a mechanism by which these regions exert control that prevents the further propagation of such seizures and returns the system to baseline. Clearly, more intensive research is needed to validate such hypotheses. As presented above, connectivity is related to the magnitude of GC. In future studies, the phase of GC values will also be considered especially to investigate desynchronization of brain regions.

As with technique, there may be limitations to this approach as well. While dipole source localization models can localize EEG activity to subcortical regions of the brain, activity that emanates from the brain stem, for example, may be more difficult. To the degree that seizure propagation in certain cases may be impacted by this, this technique may fall short in appreciating this. This should certainly be investigated in future work related to this model.

The value of epilepsy research is not only in understanding these processes but also using this information to enhance treatment. Such techniques are practical and can be used in the clinical environment. As a result, it has the potential to lead to advances that may aide therapeutic intervention. It is possible that this could aide in more precise surgical intervention (Stefan and Lopes da Silva, 2013), targeting medication treatment and neuromodulation techniques (Frye et al., 2013). We recommend that more research efforts be directed toward these aims. The implication of GC or any other causality methods into the clinical domain represents a promising means to understand and investigate neuronal information flow. These strategies may be helpful in examining brain network hubs, flexibility, and adaptability across brain regions. Nevertheless, we are still at the very early stages of understanding and measuring these processes. There is a reason to believe that is worth investigating further the accuracy and stability of biomarkers, detection of neurodevelopmental disorders, and processes involved in seizures to work toward their resolution.

## Conflict of Interest Statement

The Review Editor Rex Cannon declares that, despite having collaborated previously with the authors Robert Coben and Iman Mohammad-Rezazadeh, the review process was handled objectively and no conflict of interest exists. The authors declare that the research was conducted in the absence of any commercial or financial relationships that could be construed as a potential conflict of interest.
